# Pancreatic solitary and synchronous metastasis from breast cancer: a case report and systematic review of controversies in diagnosis and treatment

**DOI:** 10.1186/1477-7819-12-2

**Published:** 2014-01-05

**Authors:** Carlo Molino, Carmela Mocerino, Antonio Braucci, Ferdinando Riccardi, Martino Trunfio, Giovanna Carrillo, Maria Giuseppa Vitale, Giacomo Cartenì, Guido De Sena

**Affiliations:** 1 Department of General Surgery and Breast Unit, Cardarelli Hospital, Naples, Italy; 2 Department of Oncology, Cardarelli Hospital, Naples, Italy; 3 Department of Pathology, Cardarelli Hospital, Naples, Italy

**Keywords:** Breast cancer, Lobular carcinoma, Pancreatic metastasis, Synchronous metastasis, Mammaglobin

## Abstract

**Background:**

Metastases from breast cancer cause the frequent involvement of lung, bone, liver, and brain, while the occurrence of metastases to the gastrointestinal tract is rare, and more frequently discovered after a primary diagnosis of breast cancer. Solitary pancreatic metastases from breast cancer, without widespread disease, are actually unusual, and only 19 cases have been previously described; truly exceptional is a solitary pancreatic metastasis becoming evident together with the primary breast cancer.

**Case presentation:**

A 68-year-old woman reported general fatigue, lethargy, and jaundice. Abdominal ultrasound (US) and magnetic resonance imaging (MRI) showed an ampulloma of Vater’s papilla; moreover, a neoplastic nodule in the left breast was diagnosed. She underwent surgery for both breast cancer and ampulloma of Vater’s papilla. Pathological examination of pancreatic specimen, however, did not confirm primary carcinoma of the duodenal papilla, but showed a metastatic involvement of pancreas from lobular breast cancer. Immunohistochemistry has been essential to confirm the origin of the malignancy: hormone receptors and mammaglobin were expressed in both the primary breast tumor and the pancreatic metastasis.

**Conclusions:**

This is one of the few reported cases in literature of an isolated and synchronous pancreatic metastasis from breast cancer, where the definitive diagnosis was obtained only after surgery. We discuss the controversies in this diagnosis and the choice of correct treatment. The surgical resection of solitary metastases can be performed in the absence of disseminated disease.

## Background

Breast cancer is the most common malignancy in women [1,2] and metastatic spread causes frequent involvement of lung, bone, liver, and brain [3]. The occurrence of metastases from breast cancer to the gastrointestinal tract and to the pancreas is rare, and more frequently discovered after a primary diagnosis of breast cancer [4,5]. Metastatic spread to the pancreas from different primary is unusual and accounts for approximately 2% of pancreatic malignancies [6,7], while metastatic involvement of pancreas from primary breast cancer is a rare event. In a large autopsy series, the prevalence of pancreatic metastases has been described to be as high as 6% to 11% [8-10]. Although many patients with pancreatic metastases have widespread disease, isolated metastases to the pancreas can be found [11]. If the metastatic involvement of pancreas from breast cancer is unusual, detection of isolated and synchronous pancreatic metastasis is a really exceptional event, and when an isolated metastasis in the pancreas becomes symptomatic, it is often misdiagnosed as primary pancreatic adenocarcinoma. In spite of the development of imaging modalities, the preoperative diagnosis of pancreatic malignancy is still suboptimal. Image-guided fine-needle aspiration (FNA) is an accurate and safe method for evaluating pancreatic lesions, without open biopsy or laparotomy. Here we present one of the few reported cases of synchronous and solitary metastasis to the head of the pancreas from lobular breast cancer, with the purpose to review the clinical presentation, the diagnostic procedures, and therapeutic management.

## Case presentation

**Figure 1:**
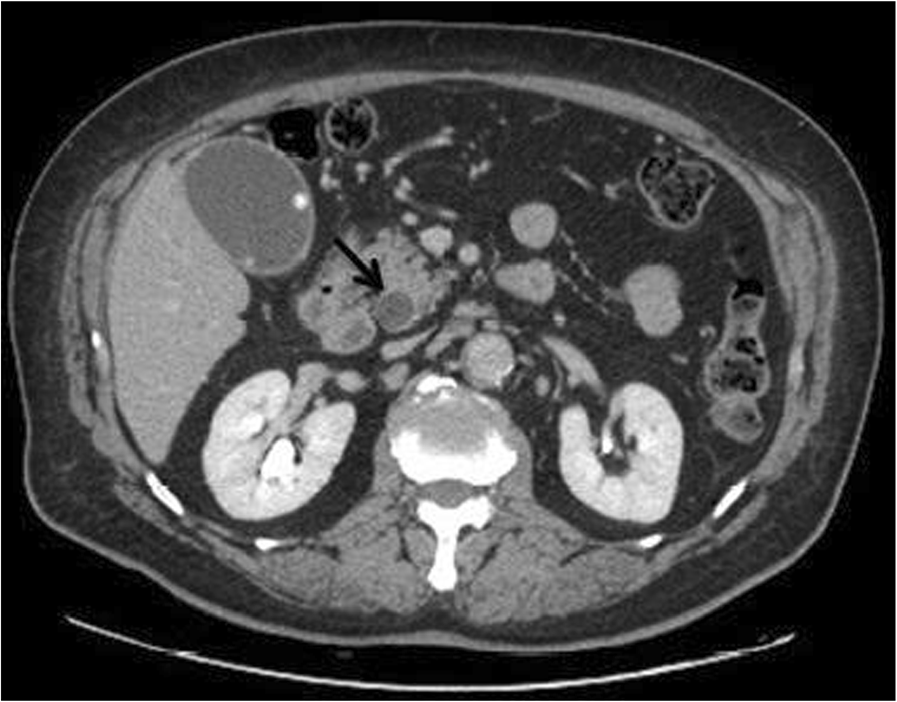
**CT scan at the diagnosis.** Contrast CTscan of the abdomen demonstrating an enlargement of the common bile duct (14 mm in diameter, see black arrow) and of the of the gallbladder with an inhomogeneous material into the lumen compatible with biliary sludge and microlithiasis. The extra-hepatic bile ducts swelling was evident until the upper papillary tract.

**Figure 2:**
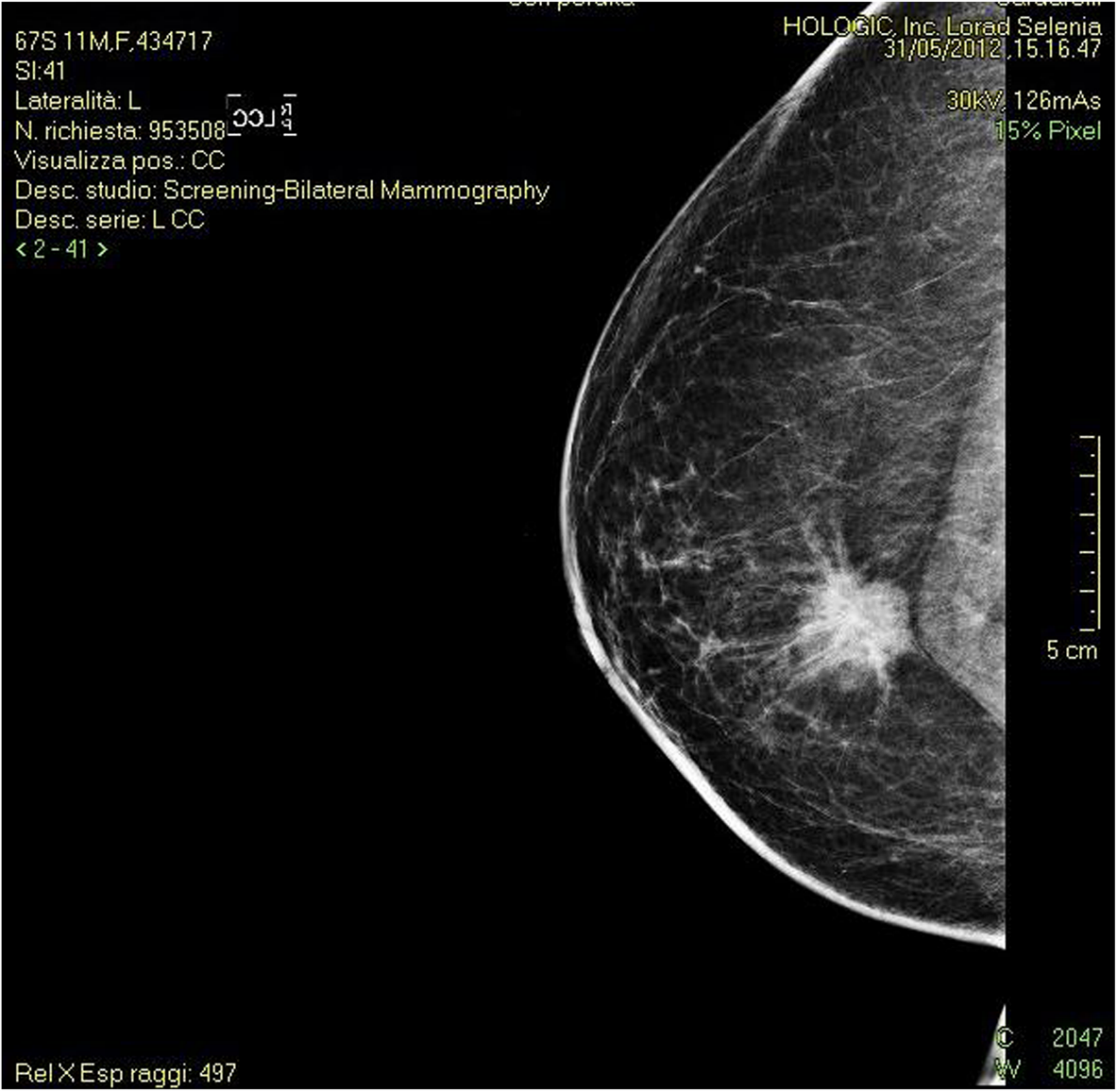
**Left mammogram.** A nodule with irregular margins revealed in the left breast.

In May 2012 a 68-year-old woman complaining of general fatigue, lethargy, and jaundice presented to the Emergency Unit of Cardarelli Hospital. Biochemical tests revealed hyperglycemia (483 mg/dl), elevated total bilirubin (4.22 mg/dl, with conjugated bilirubin of 3.49 mg/dl), high levels of CA 15.3 (320.6 UI/mL), and CA 19.9 (490.7 UI/mL), while levels of CEA and CA125 were within normal limits. Abdominal ultrasonography (US) showed biliary sludge and bile duct dilation, and magnetic resonance imaging (MRI) with cholangiography confirmed an enlargement of extra-hepatic bile ducts (14 mm in diameter) evident until the upper papillary tract, where a stop of the signal suggested a tumor of the duodenal papilla (Figure 1). Blurring of the uncinate process of the pancreas and several lymph adenopathies were reported. In a few days the jaundice increased and biochemical tests underlined a liver dysfunction (GOT 200 UI/L, GPT 238 UI/L, ALP 320 UI/L, GGT 1,441 UI/L, and cholinesterase 2,540 UI/L). Moreover, during the diagnostic procedures, a mammary US revealed a hypoechoic nodule of 25 mm of the left breast with fringed margins. After mammography with FNAC describing a 30 mm breast carcinoma in the left breast (Figure 2), she was admitted at our department of General Surgery and Breast Unit, with a diagnosis of primary breast cancer and primary carcinoma of the duodenal papilla. Total body CTscan confirmed the previous findings and didnot show other secondary involvements. An ERCP showed an ulcerate papilla, assuming the lesion as an ampulloma, but biopsies were not significant for diagnosis.

In June the patient underwent one-time surgery for both breast cancer and ampulloma of Vater’s papilla. During the pancreatic time the gland showed features of infiltrations and intraoperative Trucut biopsies were performed; the confirmation of the presence of poorly differentiated carcinoma suggestive for pancreatic origin allowed us to perform Whipple’s procedure. The breast time consisted of Patey’s mastectomy for an invasive lobular carcinoma localized in the upper inner left quadrant, measuring 3.2 cm at its longest dimension. None of the 14 axillaries lymph nodes removed were malignant.

**Figure 3:**
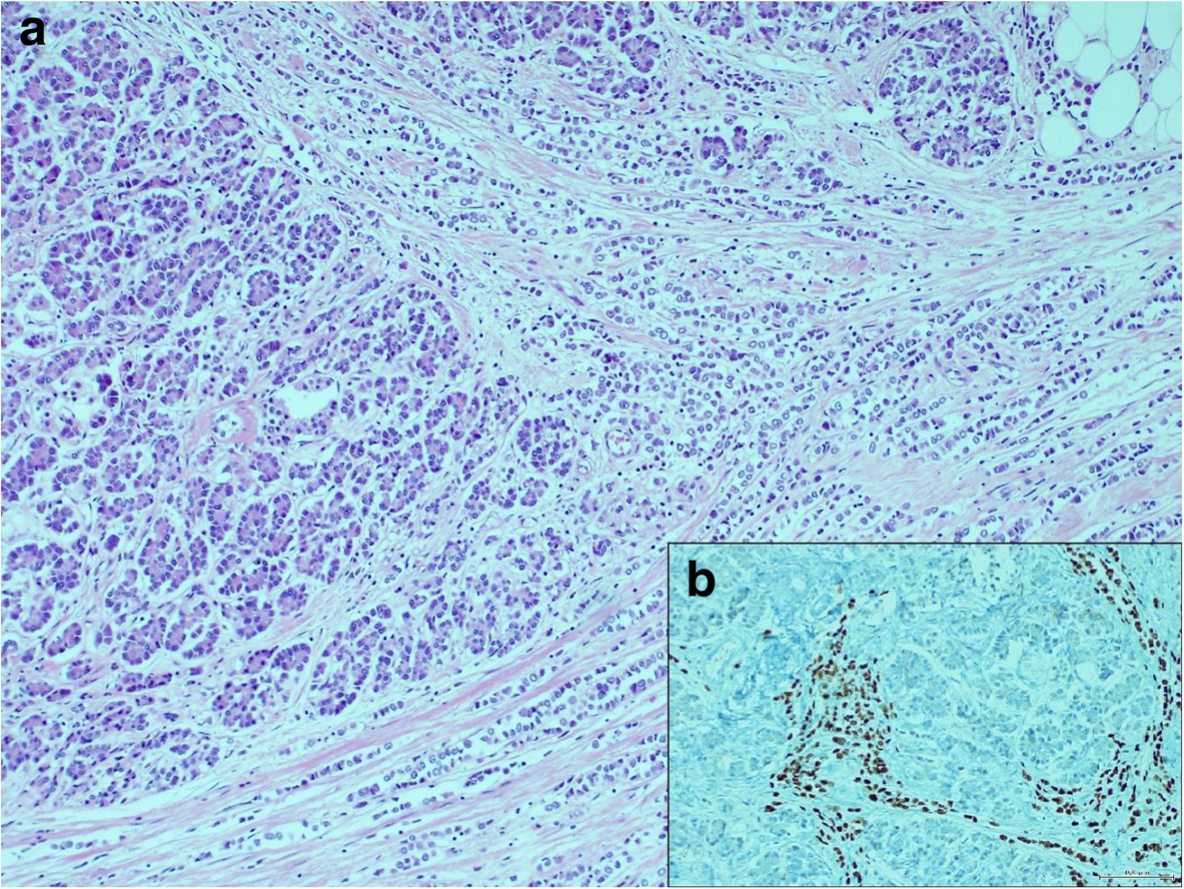
**Histological features of pancreatic mass. (a)** Small disaggregated tumor cells infiltrating the pancreatic parenchyma (H&E staining, ×100). **(b)** Immunohistochemical staining of ER in the pancreatic head mass (×250).

Pathological examination of the pancreatic mass revealed a lesion of 2.5 cm infiltrating the ampulla and the perivisceral adipose tissue, but surprisingly did not confirm primary adenocarcinoma of the pancreas: microscopic features consisted of a metastatic lobular carcinoma of the breast (Figure 3). Surgical margins of the pancreatic resection were free from neoplastic infiltration as were the isolated lymph nodes.

**Figure 4:**
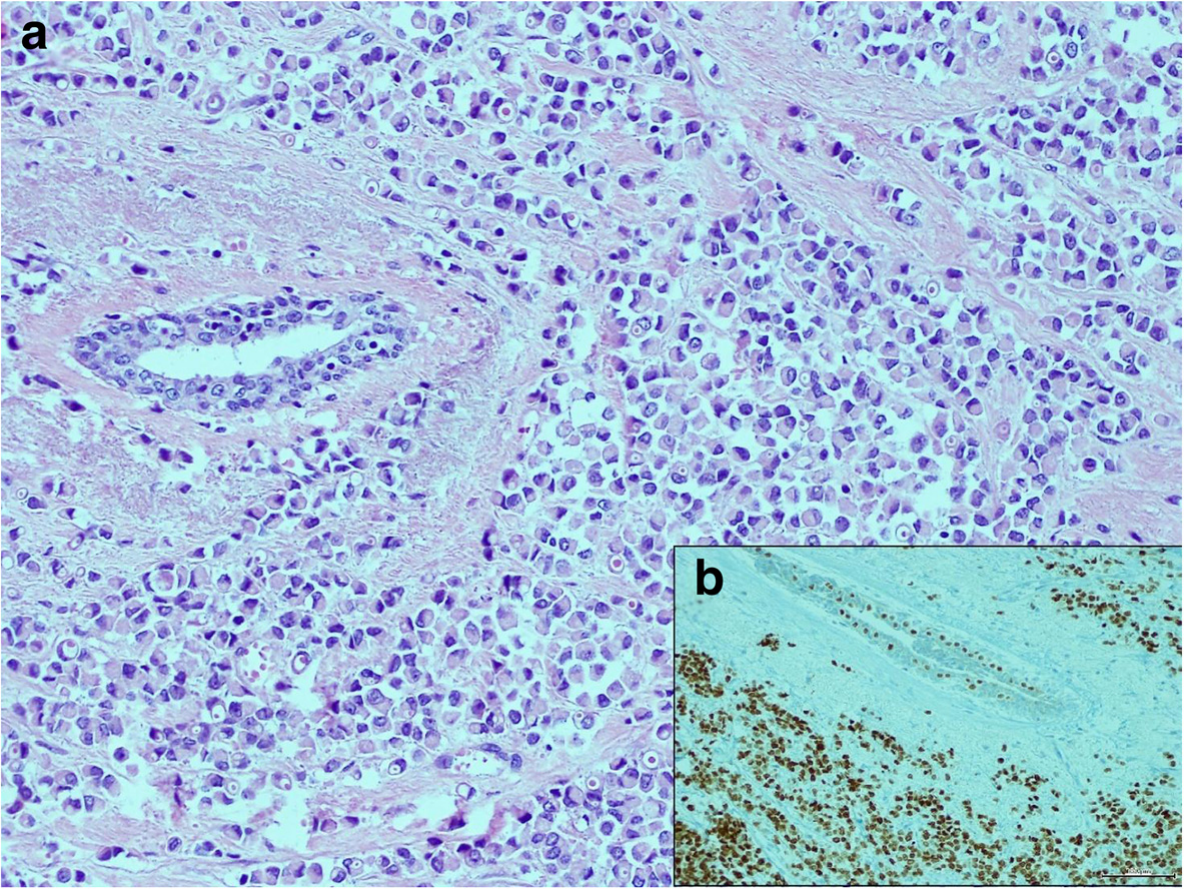
**Histological features of breast carcinoma. (a)** Breast lobular carcinoma (H&E staining, ×250). **(b)** Immunohistochemical staining of ER in the breast lobular carcinoma (×250).

Breast specimen confirmed a grade II invasive lobular carcinoma (Figure 4) exhibiting high levels of estrogen (90%) and progesterone receptors (80%), HER2*-*negative*,* ki-67 = 20%. Because of the rarity of metastases to the pancreas from breast cancer, a specific immunohystochemical panel was performed to confirm the origin. Mammoglobin was positive, hormone receptors were highly expressed, both in the breast and the pancreatic tumors, confirming secondary pancreatic involvement from lobular carcinoma.

The breast tumor board planned a first-line hormonal therapy for advanced disease with Letrozol, on an outpatient basis. Cytotoxic chemotherapy was not prescribed, while radiotherapy was not indicated. Twelve months after the operation, the patient is alive without evidence of distant metastasis confirmed by total body computed tomography (CT) scan.

## Discussion

### Clinical presentation

The metastatic involvement of the pancreas is most often related to other intra-abdominal malignancies [7], including clear cell kidney cancer, colon cancer, and gastric cancer [12,13]. A report of 1,000 autopsied cases has revealed that for breast cancer the metastatic pancreatic involvement is a rare occurrence ranking at approximately 6% to 11% of all malignancies [5,14]. Metastatic involvement of the pancreas from primary breast cancer as solitary metastasis sites has an incidence lower than 3%: the literature reflects the rarity of this clinical scenario with 23 published reports of solitary pancreatic metastases originating from breast cancer (Table 1).

**Table 1:** Clinical features of the patients with pancreatic metastases from primary breast cancer

**Author and reference**	**Patients with pancreatic metastases (*n *)**	**Patients with primary breast cancer (*n *) and subtype**	**Age (years)**	**Disease--free interval (months)**	**Presenting symptoms**	**Localization of metastases at diagnosis**	**Characteristics of metastasis at diagnosis**	**Clinical management**	**Outcome (overall survival in months)**
Akashi [15]	15	1 (lobular)	47	41	NR	Head of pancreas	Solitary	Pancreaticoduodenectomy	28
Bonapasta [16]	1	1 (ductal)	51	24	Jaundice, pain	Head of pancreas	Solitary	Cephalic pancreaticoduodenectomy	36
Azzarelli [17]	1	1 (lobular)	49	43	Jaundice	Head of pancreas	Solitary	Pancreaticoduodenectomy, RT	72
Bednar [18]	2	1 (lobular)	75	96	Jaundice, pain	Head of pancreas	Solitary	Pancreaticoduodenectomy	48
		1 (phyllodes)	57	48	Abdominal pain	Head of pancreas, lung	Widespread disease	CHT	15
Crippa [19]	13	1 (lobular)	46	60	Jaundice	Head of pancreas	Solitary	Pylorus preserving pancreaticoduodenectomy	22
		1 (lobular)	70	36	Jaundice, pain	Head of pancreas	Solitary	Pylorus preserving pancreaticoduodenectomy	38
		1 (lobular)	57	84	Jaundice, pain	Head of pancreas	Solitary	Pylorus preserving pancreaticoduodenectomy	26
Dar [20]	5	1(ductal)	76	108	NR	Pancreas, liver	Widespread disease	Palliative bypass	6
Engel [21]	1	1 (signet-ring cells)	59	46	Pruritis, dark urine	Head of pancreas	Solitary	By-pass, CHT	15
Kitamura [22]	1	1 (ductal)	55	117	Jaundice	Head of pancreas	Solitary	Percutaneous transhepaticcholangio-drainage	1
Le Borgne [23]	12	1 (lobular)	48	Synchronous	Jaundice	Head of pancreas	Solitary	Pancreaticoduodenectomy, CHT	12
Mehta [24]	1	1 (comedo type)	30	36	Jaundice, pruritis	Head of pancreas	Solitary	Pancreaticoduodenectomy, CHT, HT	27
Mountney [25]	1	1 (lobular)	57	16	Jaundice	Head of pancreas	Solitary	By-pass, HT	24
Moussa [26]	22	1 (ductal)	53	132	Acute pancreatitis	Head of pancreas	Solitary	RT, CHT, HT	50
		1 (lobular)	35	45	Abdominal mass	Body of pancreas	Solitary	Total pancreatectomy, CHT	7
Nomizu [27]	1	1 (lobular)	46	80	Jaundice	Head of pancreas	Solitary	Pancreaticoduodenectomy, CHT, HT	18
Pan [28]	6	1 (lobular)	59	182	Jaundice	Head of pancreas	Solitary	CHT, HT	21
Pappo [29]	1	1 (lobular)	52	24	Jaundice	Pancreas, gallbladder	Widespread disease	Bypass, HT	16
Pérez Ochoa [30]	2	1 (lobular)	60	1	Jaundice	Head of pancreas,	Widespread disease	Biliary stent, cephalic	2
						Bone		Pancreaticoduodenectomy, CHT	
		1 (ductal)	55	108	None	Tail of pancreas	Solitary	Distal pancreatectomy, splenectomy, CHT	2
Tohnosu [31]	1	1 (scirrhous type)	54	52	None	Tail of pancreas	Solitary	Distal pancreatectomy, CHT, HT	5
Z’graggen [32]	10	1 (lobular)	NR	96	Jaundice	Head of pancreas	Solitary	Biliary and gastric bypass (hepaticojejunostomy and gastrojejunostomy), CHT	54

CHT, chemotherapy; HT, hormonal therapy; NR, not reported; RT, radiotherapy.

Metastases may be single or multiple, synchronous or metachronous, sometimes occurring very late. Literature describes an interval time from the first diagnosis and recurrence varies from a few months to several years and considers the synchronous presentation as an extremely rare finding [33]. Cancer metastatic to the pancreas usually develops late in the course of the disease and is associated usually with widespread metastases [34,35]. Although pancreatic metastases may clinically mimic primary pancreatic adenocarcinoma [36,37], most patients (50% to 83%) with pancreatic metastases are completely asymptomatic and the pancreatic mass is detected on routine follow-up examination [38,39]. Therefore, the presenting symptoms of these specific metastases are not necessarily distinguishable from other types of pancreatic cancer, but the diagnosis should be considered in any patient with a pancreatic mass and a history of breast cancer [40].

### Diagnosis

The lack of clinical symptoms requires the use of the imaging. Most pancreatic metastases are discovered on a CT examination performed during the follow-up of patients with history of primary malignancy [41]. The introduction of contrast-enhanced ultrasonography (CEUS) has improved the diagnostic capabilities of US [42]. In the last decade new diagnostic possibilities for tumors of the pancreas derive from the FDG-PET/CT [43]. Although its role in the diagnostic management of metastases to the pancreas is not well-defined, PET appears particularly useful to exclude other metastatic sites. However, the FDG-PET/CT is not able in the evaluation of the surgical resectability of malignant lesions.

In most cases the diagnosis of secondary pancreatic tumors along with the differential diagnosis of primary pancreatic adenocarcinomas can still be difficult. Many studies suggest the possibility of a fine-needle biopsy to establish the pathological diagnosis, although some physicians believe that this procedure should be avoided in cases of resettable pancreatic masses. The use of immunocytochemistry, when available, may be useful to confirm a suspected diagnosis [44-46].

In selected patients with pancreatic metastases, surgical resection can be considered the standard of care and may largely ameliorate the survival of the patients. Data from the literature indicate that an improved survival can be achieved in patients with renal, breast, and colon carcinomas and sarcomas as primary malignancy, while patients with melanoma and lung cancer are related with a poor outcome and should be treated non-operatively [47-49]. Even in patients not amenable to surgery, a definitive tissue diagnosis can be helpful in evaluating the possibility and type of chemotherapy. In these cases and in controversial diagnostic cases, CT can be considered as an important tool in providing guidance in order to obtain a definitive tissue diagnosis [50-52].

Immunohistochemistry (IHC) has a key role in the diagnosis of mammary disease and also to discriminate metastatic breast cancer from primary pancreatic carcinoma [53-56]. Differences in Cytokeratin (CK) profile and molecular weight may aid to distinguish ductal from lobular carcinomas [57-60], in primary as in metastatic sites.

Approximately 75% to 80% of human breast tumors express hormone receptors (HRs), the estrogen receptor (ER), and/or the progesterone receptor (PgR). In a large study of 5,993 breast cancers, the positive rate for ER was noted to correlate with nuclear grade of the tumor [61].

Mammaglobin is a recently described marker of breast differentiation [62-64]: its expression has been reported in 70% to 80% of primary and metastatic breast tumors and overexpression in breast cancer tissues is associated with an unfavorable prognosis [65]. Moreover, because its expression is not altered at the metastatic site, mammaglobin may aid in the identification of breast carcinomas presenting in metastatic spread [62,66], while changes in ER, PgR, and HER2 status have been described in a significant number of patients over the course of disease progression [67].

### Treatment

The vast majority of metastatic breast cancers are incurable; hence, the primary goals of systemic treatment are prolongation of survival, alleviation of symptoms, and maintenance or improvement in quality of life [68,69].

In HR-positive and HER-2-negative disease, endocrine therapy is the treatment of first choice independent of metastatic site, unless rapid response is needed. Limited visceral metastases are not a contraindication for endocrine therapy. The choice of endocrine agent should be based on menopausal status, co-morbidities, agents received in the adjuvant setting, and the drug safety profile. If the disease progresses rapidly (within a few months) following initiation of first-line endocrine therapy, chemotherapy is generally recommended as a second-line. After chemotherapy response stabilizes (usually 4 to 6 months), a maintenance endocrine therapy can be considered [70].

Surgery can be considered a first choice of treatment not only to differentiate between metastasis and primary pancreatic carcinoma, but also to enable survival given the improved outcome with pancreatic resection in recent decades [71,72]. Resections of pancreatic metastasis account for less than 5% of all pancreatic resections [73]; in fact, only few patients with isolated disease to the pancreas should be considered for surgical resection.

Several studies [11] have demonstrated that the resection of both pancreatic and limited extrapancreatic mass can be performed with low risk for the patient, as confirmed by our case. Although the role of pancreaticoduodenectomy is not clearly defined in the management of metastatic lesions, it seems to be associated with improved survival and useful palliation. Therefore the resection of an isolated metastasis to the pancreas seems to be an advantageous element for prognosis respect to the resection of a primary tumor of the pancreas, with a 5-year survival rate of 15% to 20% compared to a median survival after successful resection of approximately 12- to 19 months, respectively [12].

## Conclusion

We described a very rare case of a patient with primary breast cancer and a misdiagnosed synchronous primary carcinoma of the duodenal papilla. After the preoperative diagnosis, our patient was treated with a surgical approach. On the basis of the preoperative findings, we performed a pancreaticoduodenectomy followed by a mastectomy to treat two synchronous primary cancers. Only subsequent pathological examinations of surgical specimens revealed that the finding in the pancreas and duodenum was actually a solitary metastasis by a lobular breast cancer.

The peculiarity of our report is that it is one of the few cases published in the literature of an isolated and synchronous pancreatic metastasis from breast cancer. Usually, the interval time from first diagnosis and recurrence varies from a few months to several years, and synchronous presentation is an extremely rare event. Table 1 shows the clinical features of the patients with pancreatic metastases from different malignancies. Among those, 23 patients exhibited metastatic involvement of the pancreas from primary breast cancer, but in 19 of these patients the metastasis was a solitary secondary lesion, without widespread disease, becoming evident several months after the primary. Only in one case, interestingly, a solitary pancreatic metastasis became evident together with the primary breast cancer, that is to say ‘synchronous’; therefore, our case seems to be the second reported in the literature.

The analysis of the current data shows that a radical surgery of both primary malignancy and its metastasis is a possible therapeutic approach for breast carcinoma associated to an isolated pancreatic metastasis, although in most cases clinical presentation of pancreatic lesions occurs only when the primary disease is in advanced stage.

## Consent

Written informed consent was obtained from the patient for publication of this case report and any accompanying images. A copy of the written consent is available for review by the Editor of this journal.

## Competing interests

The authors declare that they have no competing interests.

## Authors’ contributions

FR, AB, and CMoc drafted the manuscript. MGV, MT, GC, GC, CMol, and GDS helped to review the literature and to draft the manuscript. All authors read and approved the final manuscript.

## Authors’ information

FR, MT, and GC are specialists at the Breast Unit of Cardarelli Hospital. GDS is Head of Department of General Surgery and GC is Head of Department of Oncology. MGV is a student of the Postgraduate School of Medical Oncology at the SUN (Second University of Naples), in application to the Cardarelli Hospital.
